# Still rocking in the structural era: A molecular overview of the small multidrug resistance (SMR) transporter family

**DOI:** 10.1016/j.jbc.2022.102482

**Published:** 2022-09-12

**Authors:** Olive E. Burata, Trevor Justin Yeh, Christian B. Macdonald, Randy B. Stockbridge

**Affiliations:** 1 Program in Chemical Biology, University of Michigan, Ann Arbor, Michigan, USA; 2 Program in Biophysics, University of Michigan, Ann Arbor, Michigan, USA; 3 Department of Molecular, Cellular, and Developmental Biology, University of Michigan, Ann Arbor, Michigan, USA

**Keywords:** multidrug transporter, membrane transport, structure-function, substrate specificity, evolution, quaternary ammonium cations, SLC35, guanidinium, polyamine, flippase, EM, electron microscopy, GEBA, Genomic Encyclopedia of Bacteria and Archaea, TM, transmembrane

## Abstract

The small multidrug resistance (SMR) family is composed of widespread microbial membrane proteins that fulfill different transport functions. Four functional SMR subtypes have been identified, which variously transport the small, charged metabolite guanidinium, bulky hydrophobic drugs and antiseptics, polyamines, and glycolipids across the membrane bilayer. The transporters possess a minimalist architecture, with ∼100-residue subunits that require assembly into homodimers or heterodimers for transport. In part because of their simple construction, the SMRs are a tractable system for biochemical and biophysical analysis. Studies of SMR transporters over the last 25 years have yielded deep insights for diverse fields, including membrane protein topology and evolution, mechanisms of membrane transport, and bacterial multidrug resistance. Here, we review recent advances in understanding the structures and functions of SMR transporters. New molecular structures of SMRs representing two of the four functional subtypes reveal the conserved structural features that have permitted the emergence of disparate substrate transport functions in the SMR family and illuminate structural similarities with a distantly related membrane transporter family, SLC35/DMT.

From atomistic descriptions of membrane transport mechanism to global spread of multidrug resistance over the last century, small multidrug resistance (SMR) proteins have provided broad insights along multiple research fronts since the family’s discovery in the mid 1990s (1, 2). With just four transmembrane helices and ∼100 residues, SMR proteins are among nature’s smallest membrane transport proteins, making them ideal systems for biochemical and biophysical investigation. These same properties impeded high resolution structural characterization for many years, however, since the proteins are almost entirely embedded in the membrane, too small for cryo-EM, and with little polar surface area to form crystal contacts. Recently, new high resolution crystal structures have been determined for two functionally distinct SMR subtypes (3, 4). These complement the body of mechanistic data that have been assembled over the years and provide an opportunity to consider the molecular underpinnings of functional diversity among SMR transporters.

In general, the SMRs transport positively charged solutes across the membrane coupled to the antiport of protons (Fig. 1*A*). The resting membrane potential and pH gradient of most bacteria implies that they typically function in the active efflux of substrates. Four major functional subtypes have been described within the SMR family, and according to our bioinformatic analysis described later in this review, at least 97% of bacterial SMR genes correspond to one of these four subtypes. The first, and likely primal, SMR subtype transports guanidinium ion, a small cationic byproduct of nitrogen metabolism, and is referred to as Gdx (**g**uani**d**inium e**x**port) (5). These are also known by the name of the gene encoding them, *sugE*. The second subtype, which we refer to as Qac (**q**uaternary **a**mmonium **c**ation), are promiscuous exporters of hydrophobic cationic compounds, including quaternary ammonium antiseptics like benzalkonium and cetyltrimethylammonium (also known as cetrimonium), and polyaromatic cationic biocides like methyl viologen (also known as paraquat), acriflavine, and ethidium (1, 6, 7). The promiscuous transport phenotype of the Qac subtype gave the SMR family its name (2), and this subtype includes the well-studied multidrug exporter from *Escherichia coli*, EmrE. Associated gene names for the Qac transporters include *emrE*, *ebrA/ebrB*, *qacE*, *qacG*, *qacH*, and others. The third subtype (gene name *mdtI/mdtJ*) has been implicated in the transport of small polyamine metabolites like spermidine and putrescine (8), and the fourth subtype (gene name *arnE/arnF*) acts as a glycolipid flippase (9). In this review, we will first describe unique topological considerations shared by all four SMR subtypes, then analyze the occurrence and distribution of the different SMR subtypes among bacterial genomes. We will review recent advances in our understanding of each SMR subtype, with particular emphasis on recent high resolution structures (Table 1), and finally, analyze structural homology between the SMRs and a distantly related family of transporters, SLC35 (also known as Drug/Metabolite Transport (DMT)).

**Figure 1:**
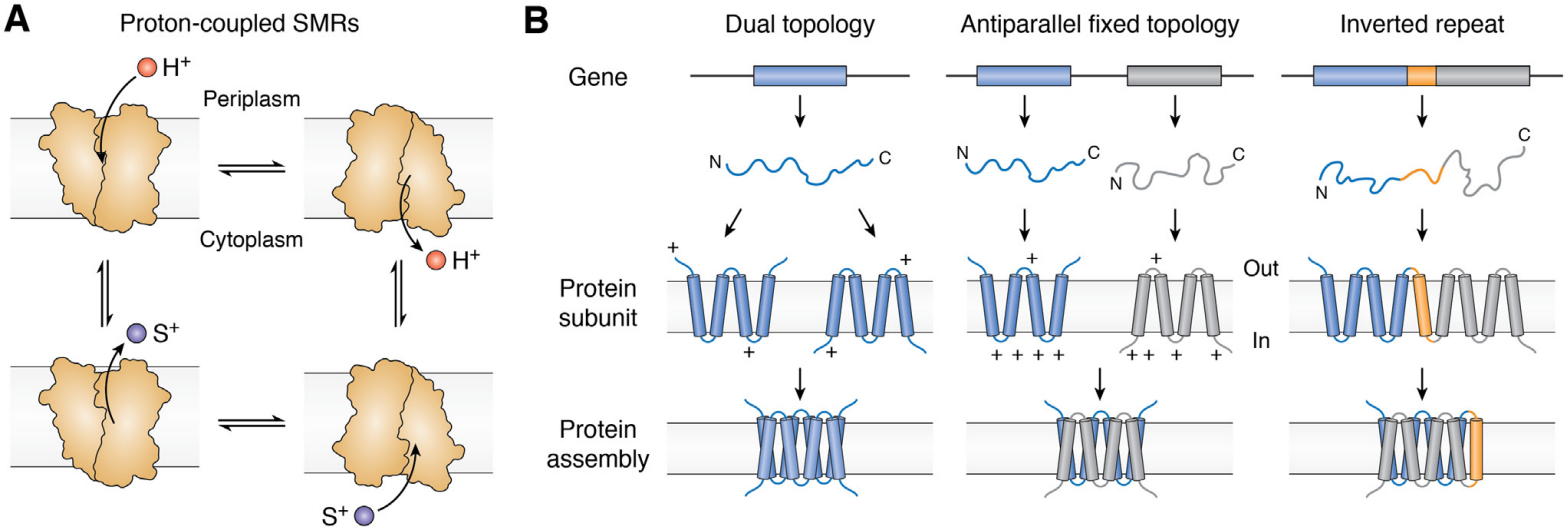
**Transport scheme and transporter topologies.***A*, simplified scheme of the transport cycle for proton-coupled SMRs. *B*, cartoons showing some potential transporter topologies. SMR proteins are found as dual topology homodimers and fixed topology heterodimers (*left* and *center*). Dual topology proteins are characterized by a balanced distribution of positively charged residues (arginines and lysines, indicated by + symbols) on the extramembrane loops and termini, whereas subunits that assemble as antiparallel fixed dimers have oppositely biased positive charge distributions. For a protein with an even number of helices, evolution of inverted repeat topology (*right*) requires the insertion of a transmembrane helix (*orange*) to enforce antiparallel architecture of fused 4-TM subunits.

**Table 1:** Structural data and models available for SMR transporters Gdx-Clo and EmrE

Protein	Substrate	Method (max. resolution)	PDB	Reference
EmrE	Tetraphenylphosphonium (TPP^+^)	Electron microscopy with 2D crystals (7.5 Å)	Data: EMD-1087 Model: 2I68	Data (12) Model (58)
Gdx-Clo	Gdm^+^	Crystallography (3.50 Å)	6WK5	(4)
Gdx-Clo	PhenylGdm^+^	Crystallography (2.53 Å)	6WK8	(4)
Gdx-Clo	OctylGdm^+^	Crystallography (2.32 Å)	6WK9	(4)
Gdx-Clo	None (pH 5.0)	Crystallography (2.32 Å)	7SZT	(3)
aEmrE_3_	None (pH 5.2)	Crystallography (2.85 Å)	7MH6	(3)
aEmrE_3_	Methyl viologen	Crystallography (3.13 Å)	7MGX	(3)
aEmrE_3_	TPP^+^	Crystallography (3.36 Å)	7SV9	(3)
aEmrE_3_	Methyltriphenylphosphonium	Crystallography (3.22 Å)	7SSU	(3)
aEmrE_3_	Benzyltrimethylammonium	Crystallography (3.91 Å)	7T00	(3)
aEmrE_3_	Harmane	Crystallography (3.91 Å)	7SVX	(3)
bEmrE S64V	Tetra(4-fluorophenyl) phosphonium/pH 5.8	NMR	7JK8	(80)
bEmrE S64V	Tetra(4-fluorophenyl) phosphonium/pH 8.0	NMR	7SFQ	(81)

Abbreviation PDB, Protein Data Bank.

a The construct EmrE_3_ bears three functionally neutral mutations, E25N, W31I, and V34M, to facilitate crystal formation (3).

b The S64V mutation preserves substrate binding but reduces the rate of conformational change by 8-fold (88).

## SMR family topology

In general, bacterial membrane proteins are inserted into the membrane according to the ‘positive inside rule’, in which the cytoplasmic face of the protein has an excess of positively charged residues arginine and lysine relative to the periplasmic face (10). The SMR proteins were among the first membrane proteins to be identified as possessing unusual “dual topology” architecture (11, 12). Dual topology proteins lack the typical biased charge distribution and are thus inserted into the membrane in both inward- and outward-facing orientations (Fig. 1*B*, left), where they can oligomerize with antiparallel subunits (13, 14). Experimental evidence suggests that individual EmrE subunits achieve their topology cotranslationally or *via* limited posttranslational annealing (13, 14, 15). The subunits can interact with each other during the immediate posttranslational protein folding stage (16, 17) but do not undergo major reorientations within the membrane after insertion to form the antiparallel homodimers (13).

In addition to dual topology homodimers, there are also numerous examples of SMR gene duplications that have given rise to co-expressed genes within a single operon that assemble as obligate heterodimers (8, 18, 19, 20) (Fig. 1*B*, middle). Sometimes called “paired SMRs” or PSMRs (7), SMRs with this arrangement are found among all four functional subtypes and likely evolved *via* multiple independent duplication events (5). In the great majority of these cases, the paired protomers exhibit opposite charge biases, which determine the orientation of each subunit in the membrane and enforces the antiparallel assembly (11).

Although dual topology and fixed antiparallel topology are only rarely observed among membrane proteins (11, 21, 22, 23), the assembly may be an evolutionary antecedent to an architecture that is extremely common among membrane transport proteins, the inverted repeat (Fig. 1*B*, right), in which a single protein possesses structurally homologous domains arranged antiparallel with respect to each other (24, 25, 26). Unlike other membrane protein families that include dual topology members (27, 28), no simple inverted repeat representatives have been detected among the SMRs, suggesting that internally fused SMR proteins might not be evolutionarily advantageous (29). Alternatively, the fusion of 4-transmembrane (TM) dual topology proteins might simply be an evolutionarily rare event, since this process requires addition of a transmembrane linker helix to connect the N and C termini of the two monomers (27) (Fig. 1*B*, right).

## Distribution of SMR genes among bacterial genomes

To gauge the distribution of SMR genes across diverse microbes, we evaluated bacterial genomes from the Joint Genome Institute’s curated set of ∼1000 Genomic Encyclopedia of Bacteria and Archaea (GEBA) genomes (30). This set of genomes was selected to reduce sampling bias and maximize phylogenetic diversity in microbial sequences used for evolutionary studies. Available sequencing data tend to be biased toward pathogenic isolates (30), and because many SMRs play a role in multidrug resistance, they are particularly prone to horizontal gene transfer *via* plasmids and other transposable sequences (4, 31, 32, 33, 34). It is therefore particularly important to use a phylogenetically representative dataset to gain a balanced view of SMR distribution among bacteria. Although archaea do possess SMR transporters (35, 36), we excluded archaea from this analysis.

**Figure 2:**
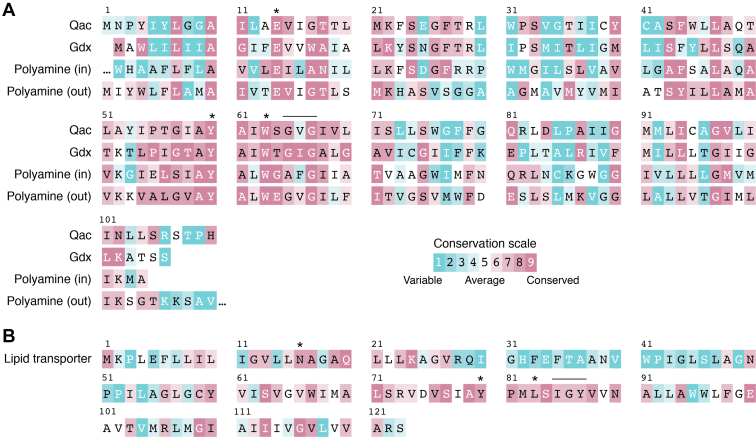
**Sequence conservation of the four major SMR transporter subtypes.***A*, alignment of representative proteins of the Gdx, Qac, and polyamine transport subtypes. Sequences from *E. coli* EmrE for Qac, *Clostridales* Gdx-Clo for Gdx, and the inward- and outward-facing polyamine transporter subunits from proteobacterium *Photorhabdus australis*. Sequences are numbered to correspond to the EmrE sequence and colored according to sequence conservation within that subtype using ConSurf (142). Highly conserved and mechanistically important residues including the central Glu, the Tyr switch, and the binding site Trp are indicated with asterisks. The TM3 GXG fulcrum motif is indicated by the *horizontal line*. *B*, representative sequence of lipid transporter from proteobacterium *Microvirgula aerodenitrificans* with sequence conservation analyzed and colored as in panel (*A*). Residues that align with the central Glu, the Tyr switch, the binding site Trp, and the GXG fulcrum are indicated.

SMR genes were identified from GEBA genomes with HMMER3.3.2 (37) using a profile Hidden Markov Model (profile HMM) constructed for the SMR family (pfam 00893). Profile HMMs for each subtype (Gdx, Qac, polyamine transport, and lipid transport) were constructed from functionally annotated clusters in a sequence similarity network of reference SMR proteins (4), and SMR sequences were assigned to the subtype that corresponded to the lowest e-value calculated by HMMR. SMR sequences were annotated “other” if the e-value was >10^−20^. Consensus sequences for each SMR subtype are shown in Figure 2, *A* and *B*, and sequence information and annotations for individual SMR proteins from this set of genomes is available for download from the Deep Blue Data repository hosted by the University of Michigan with unique identifier doi.org/10.7302/0ynd-b343.

**Figure 3:**
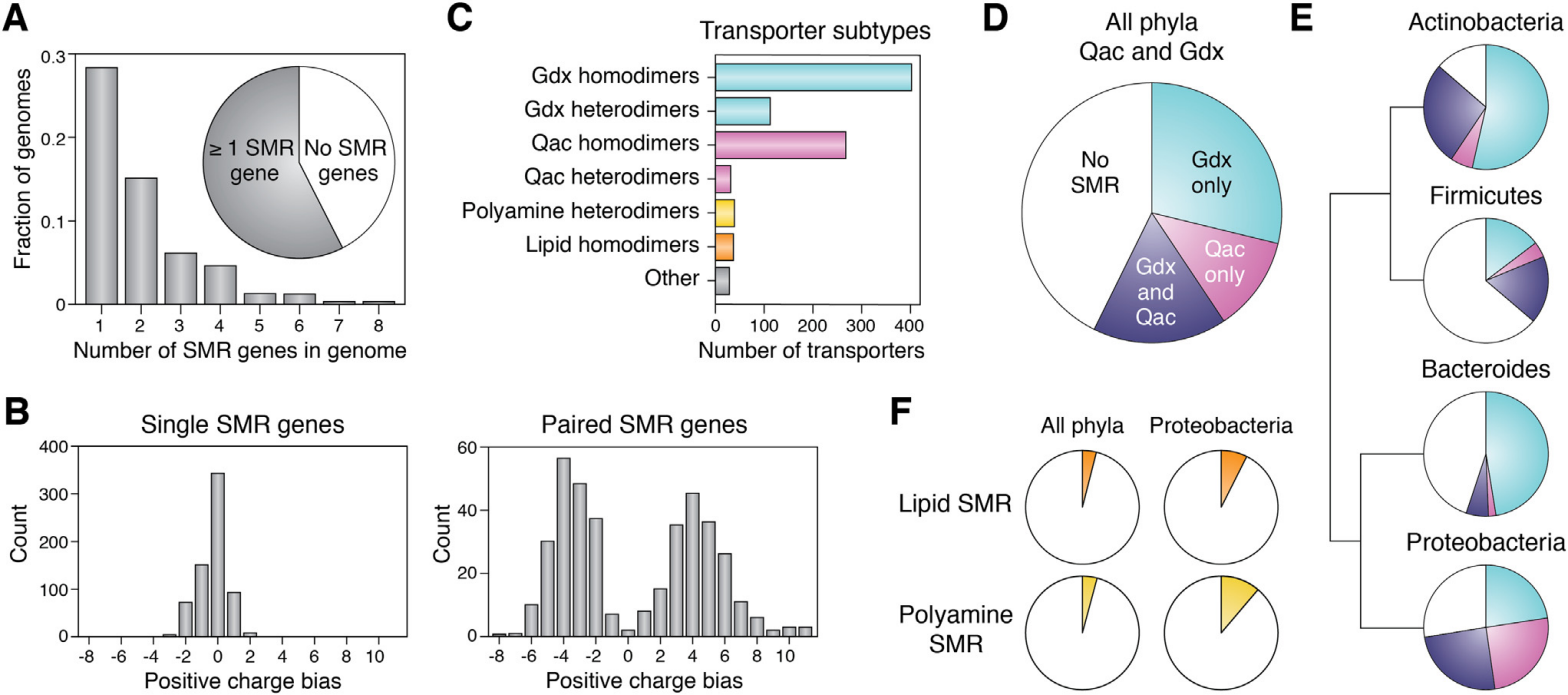
**Identification and annotation of SMR-coding genes from GEBA genomes.***A*, proportion of GEBA genomes that possess one or more SMR genes. *B*, *left*, positive charge (Arg/Lys) bias (cytoplasmic face) for SMR subunits encoded by genetic singletons (defined as no other SMR genes within 100 base pairs). *Right*, positive charge (Arg/Lys) bias (cytoplasmic face) for SMR subunits encoded by adjacent gene pairs (within 100 base pairs). The positive charge bias is given by (Arg+Lys+N-terminal amino group)_termini, loop 2_ – (Arg+Lys)_loop1, loop3_. *C*, transporter subtypes identified from GEBA genomes. Functional annotation is based on sequence comparison to functionally annotated gene clusters using HMMER (37) as described in the text. For this annotation, proteins encoded by singleton genes with unbiased positive charge distribution are annotated as homodimers, and proteins encoded by adjacent SMR genes with opposite charge biases are annotated as heterodimers. *D*, proportion of all GEBA genomes that encode Gdx, Qac, or both subtypes. *E*, frequency of genes encoding Gdx (*light blue*), Qac (*magenta*), or both subtypes (*dark blue*) for major bacterial phyla. Phylogenetic relationships and distances between clades from (143). *F*, proportion of all genomes and Proteobacterial genomes in the GEBA genome set that encode polyamine and lipid transport SMRs (*yellow* and *orange* slices, respectively). GEBA, Genomic Encyclopedia of Bacteria and Archaea.

Approximately 2/3 of the bacterial genomes from the GEBA set have at least one gene encoding an SMR protein, and ∼1/3 of the GEBA genomes encode two or more SMR genes (Fig. 3*A*). This count of genomes with multiple SMR genes reflects both paired SMR genes that encode heterodimers, as well as genomes with more than one SMR functional subtype. The majority of SMR genes have no other SMR gene within 100 base pairs, suggesting they are expressed independently. As expected for dual topology proteins (11), the Arg/Lys bias distribution for these genetic singletons is centered at 0, and only 3% encode protomers with an Arg/Lys bias greater than ±2 (Fig. 3*B*). SMR genes are also found as adjacent gene pairs. In our dataset, >95% of adjacent gene pairs encode subunits with opposite Arg/Lys biases. The Arg/Lys distributions are centered around +4 and −4 for inward- and outward-facing protomers, respectively, which assemble to form heterodimeric transporters (Fig. 3*B*). Approximately 10% of Qac transporters and ∼20% of Gdx transporters are encoded by such paired genes, as are all polyamine transporters (Fig. 3*C*). In the GEBA genome set, all the SMR lipid transporters are encoded by singleton genes, although functional pairs have been identified in some bacteria (9).

Most bacterial phyla possess genes encoding SMRs. The most prevalent SMRs are Gdx, which are found in about ∼50% of all bacterial genomes, including 80% of Actinobacteria, half of Proteobacteria and *Bacteroides*, and ∼30% of Firmicutes (Fig. 3, *D* and *E*). Qac genes are also frequent, found in ∼25% of bacterial genomes overall, including ∼50% of Proteobacteria, ∼30% of Actinobacteria, and ∼25% of Firmicutes. Many species possess both Gdx and Qac transporters. The lipid and polyamine transporters are less common and found mainly in Proteobacteria, where they are found in <10% of species (Fig. 3*F*). Thus, the SMR transporters are widespread among bacteria and dual topology Gdx and Qac transporters are the predominant SMR variants.

## Guanidinium exporters (Gdx)

Although they are the most common SMRs encoded in bacterial genomes, the Gdx transporters were also the last to be functionally annotated. The proteins were originally reported to play a role in activity of the chaperone GroEL and called SUG (**Su**ppressor of **G**roEL mutations) (38). However, this phenotype was later shown to be artefactual (7, 35, 39). With high sequence similarity to the multidrug export Qac subtype and frequent association with horizontally transferred multidrug resistance gene arrays (4, 40, 41), early characterization efforts focused on resistance to antiseptics and biocides (39, 42, 43, 44). The subtype appeared to contribute to low levels of resistance to a narrow subset of drugs, but the activity was not robust, and the proteins from this subtype remained poorly characterized until their physiological role in export of guanidinium (Gdm^+^) was established (5, 45). These proteins were renamed Gdx (Guandinium export) rather than SUG to reflect their proper functional annotation (5).

Gdm^+^ has been recognized as a byproduct of bacterial metabolism since the late 1800s, when high concentrations of Gdm^+^ were found in spoiled meats (46). But the molecular players have only begun to emerge since 2017, beginning with the discovery of riboswitch-controlled operons dedicated to Gdm^+^ metabolism and transport (45). Four unrelated classes of Gdm^+^ riboswitches have been identified (45, 47, 48, 49, 50), along with three distinct enzymatic pathways for utilizing Gdm^+^ as a nitrogen source (51, 52, 53, 54), including as a sole nitrogen source by some bacteria (53, 55). The bacteria that do not consume Gdm^+^—about half of those with Gdm^+^ riboswitches—instead produce and export endogenous Gdm^+^, likely as a metabolic waste product (5, 45). The riboswitches bind Gdm^+^ with *K*_*D*_ values between ∼60 to 200 μM (45, 47, 48, 49) to upregulate expression of the associated transporters and enzymes. These proteins have somewhat higher *K*_*m*_ values for Gdm^+^, between ∼200 μM −1 mM (4, 45, 51, 53), suggesting that Gdm^+^ accumulation becomes toxic to cells and must be mitigated within this range.

Like other SMR transporters, the Gdx harness the bacteria’s proton motive force to drive transport, exporting Gdm^+^ with strict 2:1 H^+^:Gdm^+^ antiport stoichiometry (5, 56). To prevent export of useful guanidinylated metabolites, the Gdx are highly selective for Gdm^+^ over other physiological compounds with guanidinyl moieties, such as arginine, agmatine, and creatine (5). However, electrophysiological transport experiments show that the Gdx are not exquisitely selective for Gdm^+^ either—although Gdx proteins strictly exclude guanidinyl metabolites with polar substituents, like arginine, they transport guanidinyl compounds with single hydrophobic substitutions at WT-like levels (4).

**Figure 4:**
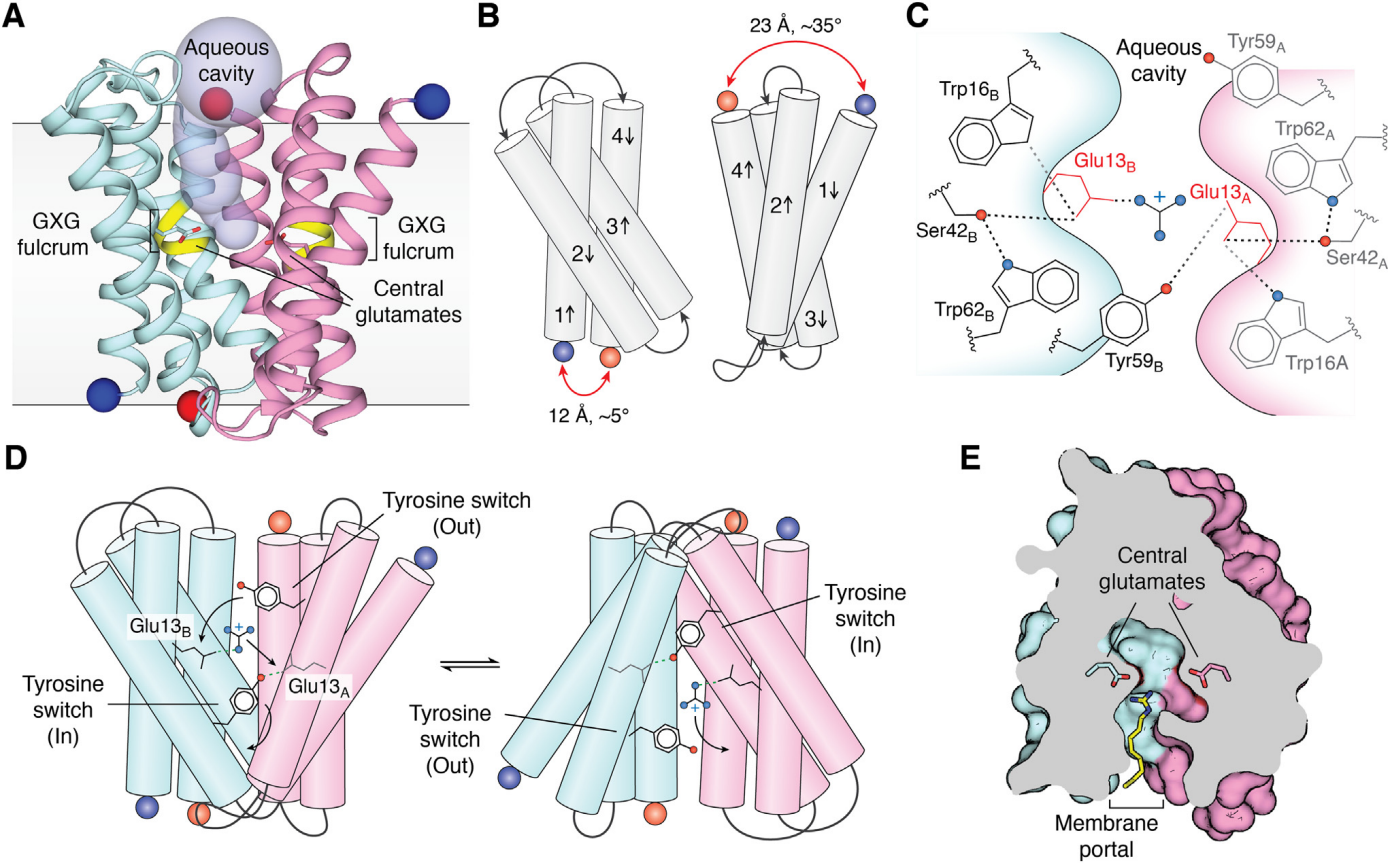
**Structure and mechanism of Gdx.***A*, crystal structure of Gdx-Clo (PDB:6WK9). Subunits are colored in *cyan* and *pink*, with N and C termini rendered as *blue* and *red* spheres, respectively. Central glutamates (E13_A_ and E13_B_ in Gdx-Clo) are shown as sticks, the conserved TM3 GXG fulcrum is colored *yellow*, and the aqueous vestibule is shown as a *light blue* surface. The approximate membrane boundaries are shown. *B*, structural comparison of individual subunits. To highlight the structural difference, the different angles between helix 1 and 4 and the different distances between the N and C termini (*blue* and *red* spheres) are indicated. *C*, diagram showing the hydrogen bond network in the substrate-binding site. *D*, proposed tyrosine switch mechanism. Rotameric movements of the tyrosine switch are indicated by *arrows* and hydrogen bonds indicated by *dashed green lines*. In this view, one of the central glutamates is occluded from view in each panel (E13_A_ in the *left panel* and E13_B_ in the *right panel*). The occluded glutamate is shown in a *lighter gray* color. *E*, top-down view of Gdx-Clo in complex with octylGdm^+^ (*yellow stick* rendering) sliced at the midpoint of the membrane (PDB:6WK9). The central glutamates are shown as sticks. The alkyl tail of the substrate extends out of the binding site through the membrane portal. PDB, Protein Data Bank.

The Gdx subtype yielded the first high resolution crystal structures from the SMR family, of a protein from *Clostridales* oral taxon 876, referred to as Gdx-Clo (4). Many of the structural features observed for this homolog had been proposed for Qac protein EmrE based on prior biophysical and biochemical experiments, establishing common structural attributes of the SMR family. Gdx-Clo possesses the expected antiparallel topology, and the two subunits assemble as an asymmetric homodimer with an aqueous cavity opened to one side of the membrane (Fig. 4*A*). Dimerization is mediated primarily by TM helix 4. The extramembrane loops also form extensive hydrogen bonded cross-subunit interactions to seal the closed side of the transporter. Each subunit of the dimer is composed of two discrete lobes delineated by a conserved Gly-Ile-Gly motif that acts as a fulcrum in TM helix 3 (TM3). The subunits differ according to a ∼35° rotation between N- and C-terminal lobes that stems from a difference in the angle of the Gly-Ile-Gly kink (Fig. 4*B*). The outward facing to inward facing conformational transition involves a structural swap between the two subunits, each changing the degree of rotation between the N- and C-terminal lobes. As a result, the inward- and outward-open conformations are 2-fold symmetric to each other, and the structural swap opens an identical, symmetry-related aqueous cavity on the opposite side of the membrane (Fig. 4*A*). This elegant mechanism for the alternating access of the substrate-binding site—a prototype for the “rocker-switch” mechanism used by many other transporters—was first predicted and demonstrated for EmrE (57, 58), and the same TM3 fulcrum motif (Gly-hydrophobic-Gly or GXG) is conserved in the Gdx, Qac, and polyamine subtypes (Fig. 2).

The substrate-binding site is located at the bottom of the aqueous cavity, with the positively charged Gdm^+^ situated between a pair of negatively charged glutamates, E13_A_ and E13_B_, one contributed by each subunit. These “central glutamates” are conserved and essential in the Gdxs, the Qacs, and the polyamine transporters (5, 8, 59). In addition to binding positively charged substrate, the central glutamates are protonatable at physiological pH and carry protons across the membrane during the opposing leg of the antiport cycle (59, 60). This common binding site for the small molecule substrate and the two antiported protons favors alternating binding site occupancy by the substrates and sets the 2:1 H^+^:Gdm^+^ stoichiometry measured for Gdx (5).

In Gdx-Clo, the central glutamates are fixed in position by a polarized stack of alternating hydrogen bond donors and acceptors, including W16, S42, and W62 (4) (Fig. 4*C*). Mutation of any one of these residues to remove H-bonding capacity substantially impairs transport function (4). In the structures, the substrate Gdm^+^ is directly coordinated by E13_B_, whereas E13_A_ is deflected away from the Gdm^+^ by a cross-subunit interaction with Y59_B_ (4). Y59_A_, in contrast, points away from the substrate-binding pocket and into the aqueous vestibule. The divergent poses of Y59 presented a mechanistic proposal for conformational change by the SMR transporters (4, 61) whereby the rotameric switch of Y59_A_ from the aqueous vestibule toward E13_B_ displaces the substrate Gdm^+^ from its interaction with E13_B_. The Gdm^+^, in turn, engages with E13_A_, displacing Y59_B_, which undergoes the converse rotameric switch, away from the central glutamates. This “tyrosine switch” has been proposed to trigger the global conformational swap that opens an aqueous cavity to the other side of the membrane, where Y59_B_ ultimately rests (Fig. 4*D*) (4). This tyrosine is almost perfectly conserved in all SMR subtypes and is mechanistically essential in all three SMR subtypes in which the effect of its mutation has been tested (3, 4, 8, 62), suggesting that the tyrosine switch is fundamental to transport by the SMRs.

In addition to the central glutamates and the tyrosine switch, the structures of Gdx-Clo revealed a third structural feature that is likely to be conserved among other SMR subtypes, the membrane portal (4). This portal is defined by TM2_A_ and TM2_B_, which form one side of the binding pocket and splay apart on the open side of the transporter. The gap between these helices is lined by hydrophobic sidechains and could, in principle, permit substrate access between the aqueous substrate-binding site and the membrane interior (Fig. 4*E*). In EmrE, spectroscopic experiments lead to the suggestion that the hydrophobic residues lining this portal act as a gate that permits access for the lipophilic substrates to the binding site (61, 63). Similar lateral openings are well-described features of both lipid and drug transport proteins (64, 65, 66), permitting hydrophobic or amphipathic substrates to diffuse between, or have simultaneous access to, the membrane and the substrate-binding pocket.

The significance of the portal for the Gdx subtype is less readily apparent, however, since the physiological substrate Gdm^+^ is small and hydrophilic and would be expected to access the binding pocket directly from aqueous solution. Nonetheless, structures of Gdx-Clo with phenyl-bound and octylGdm^+^-bound showed that these non-natural substrates utilize the membrane portal to accommodate their hydrophobic substituents, while their guanidinyl headgroups bind between the central glutamates in the binding pocket, similar to Gdm^+^ (4). These structures rationalize prior observations that Gdx transport hydrophobic, singly substituted guanidiniums (4, 5). Moreover, the positioning of these non-natural substrates also suggested a mechanism to select against natural guanidinyl metabolites (4). Should a compound such as arginine or agmatine enter the binding site in the same orientation, its polar tail would likewise be positioned to extend from the binding pocket through the membrane portal. But the hydrophobic membrane interior would not favorably interact with the polar substituents, and thus, the membrane itself could contribute to selectivity against natural guanidinylated metabolites (4). The membrane portal might also explain the association of Gdx-encoding genes with multidrug resistance gene arrays in environmental reservoirs (4, 40, 41): hydrophobic guanidinyl compounds that enter the biosphere *via* municipal wastewater or farm runoff present microbes with a persistent low-grade toxic threat (67, 68, 69) that could be mitigated by a Gdx exporter. Examples of such common biocides include the agricultural antifungal dodine (decylGdm^+^) and pharmaceuticals like metformin, which is excreted into wastewater, where it is slow to degrade and accumulates to levels of environmental concern (70, 71). Likewise, this portal may explain prior observations that cationic detergents bind to Gdx homologs (39).

## Drug and antiseptic exporters (Qac)

Transporters of the Qac subtype garnered early attention for their role in bacterial multidrug resistance. Frequently found in clinical and agricultural isolates (7, 72), this SMR subtype confers resistance to the quaternary ammonium compounds used as common hospital and household antiseptics. These antimicrobial agents were introduced in the 1930s, and evolutionary analysis suggests that it was around this time that the immediate ancestor of the clinically important vector for multidrug resistance, the class I integron, emerged (73). This ancestral class I integron likely consisted of an integron/integrase sequence to capture drug resistance genes, a transposable element to facilitate its spread among microbial populations, and a single resistance gene: an SMR transporter of the Qac subtype (73). Sequence analysis suggests that Qac SMRs have been dynamically associated with these and other drug resistance gene arrays over the last hundred years, gained and lost multiple times as these elements have spread among both pathogenic and environmental bacteria (74, 75). Today, Qac transporters remain adaptive to subinhibitory concentrations of quaternary ammonium antiseptics found in wastewater and surface runoff and remain among the most common genes isolated from human-adjacent environments (31, 32, 33, 34). By conferring this selective advantage against ubiquitous environmental biocides, the Qac SMRs coselect for other resistance genes in the cassettes that provide resistance against more potent clinical antibiotics (76), contributing to the continued spread of multidrug resistance.

**Figure 5:**
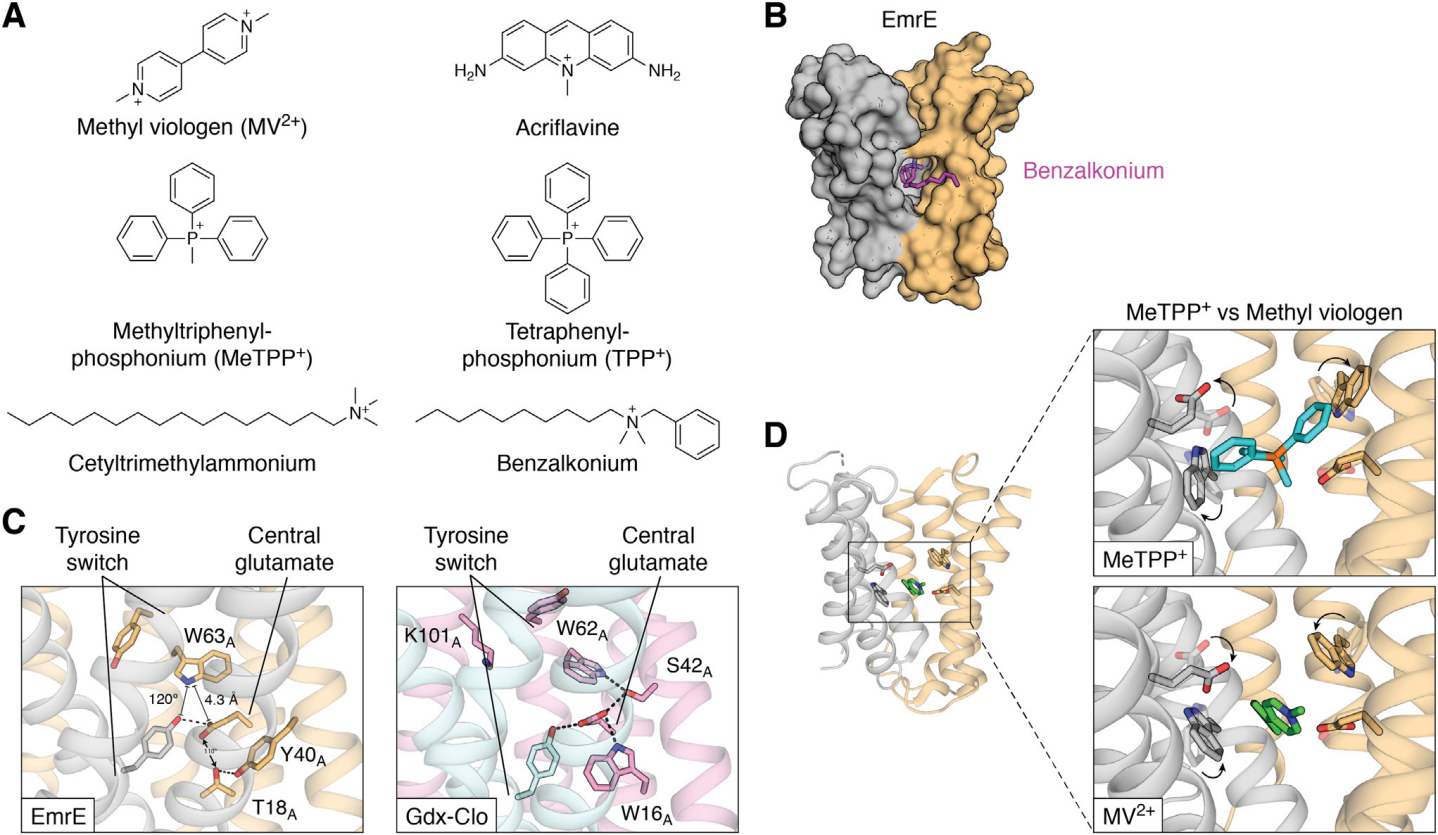
**Substrate binding to EmrE.***A*, examples of planar aromatic, quaternary ammonium/phosphonium, and alkylated substrates transported by EmrE. *B*, model of EmrE with benzalkonium bound (3). EmrE subunits in *gray* and *tan* surface rendering with benzalkonium shown as *purple* stick representation. The substrate’s alkyl tail extends from the binding site into the membrane *via* the conserved membrane portal. *C*, comparison of EmrE (PDB:7MH6) and Gdx-Clo (PDB:7SZT) substrate-binding sites with putative hydrogen bonds (distance <3.5 Å, angle 140–180°) represented as *dashed lines*. Distances/angles are shown for potential H-bond partners that possess nonoptimal geometry. *D*, EmrE substrate-binding site with different substrates bound. *Top panel*: bulky quaternary phosphonium substrate, methyltriphenylphosphonium (PDB:7SSU). *Bottom panel*: planar aromatic substrate, methyl viologen (PDB:7MGX). To aid comparison, sidechain positions in the presence of the alternative substrate is rendered as lightly colored, transparent sticks. Arrows indicate movements of the central glutamates and binding site tryptophan (W63) that accommodate the differently sized substrates. PDB, Protein Data Bank.

Meanwhile, the *E. coli* variant, EmrE, has become one of the best studied bacterial multidrug exporters over the last 25 years. EmrE was originally shown to transport a variety of polyaromatic, cationic antimicrobial compounds (1) (Fig. 5*A*). Early, low resolution electron microscopy (EM) of 2D crystals demonstrated the unusual antiparallel architecture and established an elementary understanding of the helical connectivity and protein fold (12, 58). Although high resolution structural information lagged, biochemical and biophysical studies provided a detailed molecular picture of the protein and its transport cycle. As a reference, we provide a summary of the scanning mutagenesis studies that have been performed for EmrE in Table 2. Although too extensive to discuss individually here (see reference (77) for an in-depth review of EmrE mutagenesis), these studies provided a functional grounding for the interpretation of electron paramagnetic resonance (EPR) distance measurements (63), models based on the low-resolution EM data with computationally predicted sidechains (61, 78, 79), models derived from NMR chemical shifts and substrate/protein distance restraints (80, 81), and ultimately, the high resolution crystal structures, as discussed later (3).

**Table 2:** Selected scanning mutagenesis studies of EmrE

Mutations tested	Substrates tested	Assay	Reference
Scan: all residues to Ala, Gly, and Val	Ethidium, acriflavine, methyl viologen	Bacterial resistance	(88)
Scan: all residues to Cys	Ethidium	Bacterial resistance	(85)
33 variants of mechanistically important residues	12 drugs including polyaromatics, quaternary ammonium cations, and antiseptics with alkyl tails	Bacterial resistance	(89)
18 mutations in helix 1, loop 1, and helix 2 to Cys	TPP^+^	Binding (purified, reconstituted protein)	(144)
48 mutations throughout protein to Cys	Methyl viologen, acriflavine, ethidium	Bacterial resistance	(145)
All Glycines (12) mutated to Cys, Ala, Pro	Methyl viologen, acriflavine, ethidium	Bacterial resistance	(146)
All Tyrosines (5) mutated to various residues. 18 total mutants tested.	Methyl viologen, TPP^+^	Transport and binding experiments	(62)
All Tryptophans (4) mutated to Cys.	TPP^+^, methyl viologen, acriflavine, ethidium	Transport, binding, and bacterial resistance experiments	(86)
All acidic and basic residues (10) mutated to various residues	Ethidium, acriflavine, methyl viologen	Transport and bacterial resistance experiments	(60)

These EmrE crystal structures were determined with structurally diverse substrates bound, including planar polyaromatics and tetrahedral quaternary phosphoniums and ammoniums (Table 1). The electron density determined using X-ray crystallography shows high correspondence with previous low resolution EM data obtained for EmrE in lipid bilayers (12), implying that the crystal structures represent a native, low energy conformation. The molecular details of the crystal structures also agree with specific predictions from spectroscopic and mutagenic studies (63). Recent models from the NMR experiments (80, 81) exhibit notable structural differences with the crystal structures (3) and with the available computational and EM models (58, 61). These differences, and potential reasons for the differences, are discussed in depth in (3) and will only be briefly summarized here. In the NMR models, the subunits of the dimer are arranged more parallel with respect to each other, and the loops are unpacked, permitting aqueous access to the binding site from both sides of the membrane. The hydrogen bond network in the binding site is also rearranged with respect to the recent crystal structures. It is possible that the NMR models represent functional intermediate states, such as those suggested by prior EPR experiments (63), and that the differences between the crystallography or EM density (which largely agree with each other) and NMR models and are due to differences in the experimental conditions. However, it is also important to note that the NMR models are based on relatively few experimental measurements of distances between backbone atoms and bound substrate and that the models (including sidechain placement) are generated computationally, based on these distance restraints and backbone chemical shift measurements. Since the recent crystal structures are in the best agreement with the EM maps and are the only structural models of EmrE with experimental electron density that supports sidechain placement without further computational modeling, we will focus our analysis on these crystal structures (3). Note that previous EmrE crystal structures (82) (Protein Data Bank codes 3B5D, 3B62, 3B61) are low quality and incomplete (only C_α_ atoms are modeled) and experimental maps are unavailable in the Protein Data Bank. These prior structures are broadly considered inadequate for molecular inference (3, 61, 78, 79) and should not be used.

Both the recent structures and the long history of functional data show that EmrE has many major mechanistic features in common with Gdx-Clo, including the central glutamates in the binding pocket that contribute to alternate binding of drug and protons (59, 83, 84), the tyrosine switch engaged in a cross-subunit interaction (61, 62), the GXG fulcrum that kinks TM3 and defines the N- and C-terminal lobes of each subunit (58, 85), and the hydrophobic portal that permits access between the binding pocket and the hydrophobic interior of the inner lipid membrane for hydrophobic substrates and substrate substituents (61, 63). Based on information from the crystal structures of EmrE with benzyltrimethylammonium (the headgroup of the common household antiseptic benzalkonium) and Gdx-Clo with octylGdm^+^, a model for benzalkonium binding was constructed (3), illustrating how the membrane portals of both the Gdx and Qac subtypes can be exploited to bind substrates with extended alkyl substituents (Fig. 5*B*).

Given the structural similarities and high sequence conservation, why then can EmrE bind and transport a much more diverse range of substrates than Gdx-Clo? The crystal structures in complex with substrates suggest that, despite shared sequences, EmrE and Gdx-Clo also have important structural differences. Although many of the binding pocket residues that serve as hydrogen bond donors or acceptors are conserved in EmrE, they do not form as an extensive an inter-residue H-bond network as is observed in the binding site of Gdx-Clo. Peripheral binding site residues W63 and S42 are both present, but they do not H-bond with other EmrE sidechains (4). Many potential H-bond interactions have either poor geometry or longer interaction distances than ideal (Fig. 5*C*). As a result, the central glutamates and conserved binding site Trp (W63 in EmrE) are comparatively unconstrained and able to adopt different rotamers in the presence of different substrates. EmrE’s central glutamates move closer together or farther apart to accommodate flat planar substrates or bulky quaternary compounds (Fig. 5*D*). At the same time, the binding site Trp rotates over ∼80° to stack against aromatic rings of different substrates bound in different poses (3) (Fig. 5*D*). Unlike Gdx-Clo, in which the residues that contribute to the H-bond stack stabilizing the central glutamates cannot be altered (4), many of the analogous mutations to reduce H-bonding capacity are tolerated in EmrE (3). Notably, the binding site Trp, W63, which had been shown to be essential in all previous studies with aromatic substrates (85, 86, 87, 88), is not required for the transport of nonaromatic substrates by EmrE (3, 89).

These structural observations are in accord with NMR and computational studies that suggest that EmrE possesses an unusual degree of structural plasticity that might contribute to substrate polyspecificity (79, 90). However, it should be emphasized that the observed structural perturbations are limited to the sidechains. Larger conformational changes involving the backbone are not necessary to explain the binding of diverse substrates to EmrE, and such perturbations are not observed in any of the five drug-bound crystal structures (3). Likewise, the low pH, proton-bound crystal structures of EmrE (as well as Gdx-Clo) do not exhibit major structural differences relative to the substrate-bound structures, with only local changes in the position of the central glutamates (3). However, EPR studies have suggested that upon protonation, the conformational ensemble of EmrE becomes more heterogenous than in the presence of the drug TPP^+^ (63). Thus, the doubly protonated state might not exist in a single predominant stable conformation and the reported low pH crystal structure may capture only one species in this ensemble.

Although the crystal structures provided essential insight into the molecular basis for substrate binding by EmrE, the transport of these disparate substrates poses additional problems, requiring the choreography of substrate binding and dissociation, conformational exchange, and proton antiport. Spectroscopic techniques, including NMR and EPR, have been integral to fleshing out a dynamic picture of EmrE. NMR studies in lipid membranes and in bicelles have identified various mutations that slow or eliminate conformational change but preserve substrate binding, isolating residues involved in the first process and not the second (88, 91). NMR measurements have shown that the kinetic behavior of EmrE, including the rate of conformational exchange, differs depending on the substrate, demonstrating that different substrates have different affinities for the transition state of the conformational exchange, as they do in the ground state (92). Likewise, protonation of the central glutamates accelerates conformational transition (93), perhaps reflecting the same ground state destabilization that leads to heterogeneity in the conformational ensemble upon protonation (63). The reduction of free energy of conformational transitions upon substrate binding is a classical requirement for coupled substrate antiport (94).

However, emerging evidence also suggests that under certain conditions, EmrE violates tenets of classic transport mechanisms. Conformational exchange of the apo (proton- and drug-free) and single proton–bound transporter have been reported (83, 90, 95), as has simultaneous binding of proton with some drugs such that both can be carried across the membrane together (80, 96). The conformational exchange rate of EmrE with different substrates is not tightly correlated with the rate of substrate transport, hinting that different substrates, particularly high affinity substrates, might undergo futile cycles and remain bound as the transporter transits between inward and outward open states (92). Kinetic modeling (97) suggests scenarios in which the microscopic rate constants measured for each potential binding event and conformational transition in the transport cycle combine to reduce the stoichiometry noticeably from the 2:1 H^+^:substrate stoichiometry measured for Gdx (5) and many EmrE substrates (6). These studies suggest that, in some cases, the specific proton and substrate gradients and substrate-binding energy may even lead to cycles of substrate import (96, 97).

A limitation of such free exchange transport models is that they permit potential pathways for proton leak. If the inward-to-outward facing transition of the unoccupied or singly protonated transporter is not energetically prohibitive in living bacteria, such transport cycles would contribute to the dissipation of the proton motive force. For EmrE, different mechanisms have been proposed to explain the apparent absence of detrimental leak pathways *in vivo* and *in vitro*. In one proposal, the central glutamates are electrostatically independent so that the proton cannot “hop” from the glutamate with the lower p*K*_a_ to that with the higher p*K*_a_, preventing proton release from the singly protonated state after the conformational swap (84). Alternatively, it has been suggested that the proton pathway is gated by a C-terminal histidine residue that is highly conserved among the Qac subtype, which occludes the binding pocket in the absence of drug, preventing proton leak (98). It should also be mentioned that the quaternary phosphonium substrates used in these transport experiments are not encountered by bacteria outside the laboratory. Thus, the transport properties for such anthropogenic chemicals have not been optimized by purifying selection in bacterial populations over evolutionary time. While it is possible that proton slippage and deviations from ideal stoichiometry are evolved properties of the transporter to handle diverse substrates (96), it is also possible that these mechanistic features reflect nonoptimized transport of non-native compounds and that transport of native substrates (whether the drug-like molecules produced by microbes in competitive niches or yet-unknown metabolites) is more parsimonious. Native substrates of the Qac transporters have yet to be identified, however, so this remains an open question.

## Polyamine transporters

Polyamines, such as spermidine, putrescine, and cadaverine, play myriad roles in diverse bacteria (99, 100, 101, 102). These small, charged metabolites are synthesized or taken up from the environment by bacteria, where they can be used as synthetic precursors to siderophores (103, 104) or structural components of the cell wall (105, 106), contribute to oxidative stress resistance (107, 108), or interact with nucleic acids to modulate translation (102). Polyamines also serve as signals for the induction of virulence genes (109, 110, 111) and surface behaviors like biofilm formation and swarming (112, 113, 114, 115, 116). However, excessive accumulation of polyamines is toxic (117). A subset of transporters from the SMR family has been implicated in polyamine export in *E. coli* and *Shigella* (8, 118). These proteins are proposed to serve as a “safety valve” when intracellular polyamines accumulate to toxic levels (118). The transporters form heterodimers, and their genes are always found as pairs, annotated *mdtI* and *mdtJ* in *E. coli* (8). Native expression is low and is regulated by accumulation of polyamines and bile salts (8, 118). Although biochemical information is relatively limited for the polyamine transporters, mutagenesis coupled with growth assays has demonstrated that key mechanistic residues for Qac and Gdx function, including the central glutamates, the tyrosine switch, and the binding site tryptophans, are critical for function of the MdtIJ complex (8), implying that the polyamine transporters share mechanistic similarities with the more extensively characterized Gdx and Qac subtypes.

## Lipid transport proteins

The most distantly related members of the SMR family are reported to act as glycolipid flippases. The SMR genes (annotated *arnE* and *arnF* in *E. coli* and *Salmonella enterica*) are found in larger biosynthetic operons that contribute to the chemical modification of lipid A in the outer membrane with 4-amino-4-deoxy-l-arabinose (L-Ara4N) (119). This synthetic pathway contributes to polymyxin resistance by reducing the electrostatic interactions of lipid A with the cationic polymyxin antibiotic (119). In *S. enterica* serovar Typhimurium, the role of this SMR subtype is to transport undecaprenyl phosphate aminoarabinose (9), a lipid that carries L-Ara4N from its site of synthesis in the cytoplasm to the periplasmic leaflet, on its way toward lipid A in the outer membrane (120). Deletion of the SMR transport proteins prevents localization of L-Ara4N to the outer membrane and thus prevents cells from acquiring polymyxin resistance *via* this route (9). There are indications that the *S. enterica* flippase has somewhat broader specificity, including genetic complementation of flippase deletion mutants in synthetic pathways that require transport of different glycolipids (121, 122).

The lipid SMRs are the only subtype that transports noncationic substrates, and these proteins often possess an asparagine in place of the central glutamate (Fig. 2). This replacement also suggests that lipid transport may not be proton coupled, since the central glutamates are also responsible for proton binding in proton-coupled SMRs. Because undecaprenyl phosphate aminoarabinose is synthesized in the cytoplasmic leaflet (120), transport of the lipid down its concentration gradient to the periplasmic leaflet *via* facilitated diffusion might be sufficient. However, this has not been established experimentally. A membrane portal similar to that observed in structures of EmrE and Gdx-Clo would be an obviously useful feature for lipid transport, permitting the substrate’s prenyl tail access to the membrane while the polar headgroup is ensconced within its protein-binding site, a familiar feature of other lipid flippases (123). Indeed, in this SMR subtype, the hydrophobic character of the TM2 residues is retained, and we therefore conjecture that the membrane portal is conserved as well. The tyrosine switch is also conserved in the lipid SMRs, despite their overall low sequence similarity with other SMR subtypes.

## Structural relationship between the SMR and the SLC35/DMT folds

**Figure 6:**
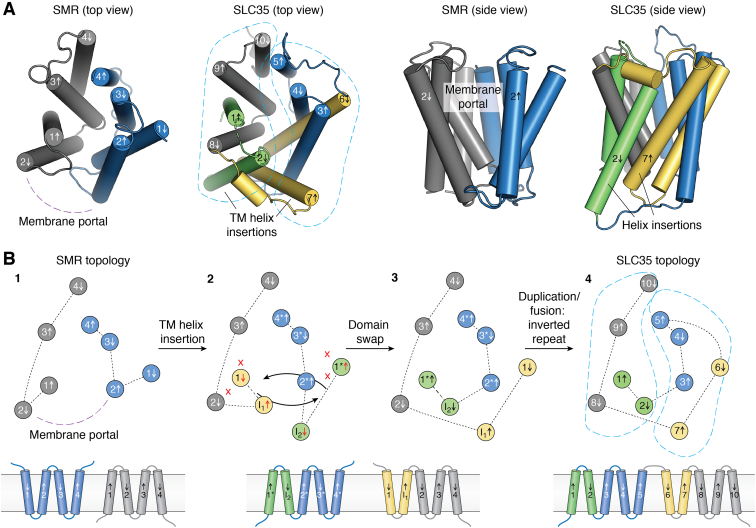
**Structural relationship between the SMR and SLC35 folds.***A*, structural comparisons of Gdx-Clo (PDB:6WK9) (4) and SLC35 CMP-sialic acid transporter (PDB: 6I1R) (125). For the SMR fold, the individual subunits are colored in *gray* and *blue* and the membrane portal is labeled. For the SLC35/DMT fold, helices involved in a potential domain swap are colored in *green* (for the *blue* monomer) and *yellow* (for the *gray* monomer). The transmembrane (TM) helix insertions are indicated. The two transport domains are outlined by the *dashed turquoise line*. *B*, a potential pathway for divergent evolution of the SMR and SLC35/DMT folds. Topology is shown as a *top-down* cartoon, as in the left panels of (*A*). Panel 1: SMR topology with membrane portal indicated. Panel 2: insertion of a TM helix (helices I_1_ and I_2_) inverts helix 1 and 1∗ with respect to the other helices in the subunit, introducing clashes and disrupting helical packing (*red* x symbols). The original packing can be restored by swapping the positions of the *yellow* and *green* helices, indicated by *arrows*. Panel 3: Dual topology ancestor of the SLC35/DMT fold possesses a helix insertion that seals the membrane portal and a domain swap involving the *yellow* and *green* helices that preserves structural homology with the SMR fold. Panel 4: a duplication/fusion event links the C terminus of the first subunit (helix 5) with the N terminus of the second subunit (helix 6) and fixes this topology in the SLC35/DMT lineage. The transport domains are outlined by the *turquoise dashed line*, as in the SLC35 structure in panel (*A*). PDB, Protein Data Bank.

Although other dual topology transporter families have representatives with inverted repeat topology (27, 28), the SMR family does not possess such internally fused transporters with detectable sequence homology (29). However, structural analysis of transporters from the DMT superfamily, which possess the SLC35/DMT fold, suggests that the SMR fold might nonetheless have been preserved by evolution as an inverted repeat. Structures of transporters with the SLC35/DMT fold, including a bacterial aromatic amino acid exporter, a protozoan drug exporter, and eukaryotic organellar sugar/nucleotide transporters (124, 125, 126, 127, 128), possess striking structural homology to the bacterial SMRs (TM helix RMSD 2.6–3.8 Å) despite sharing no sequence similarity (Fig. 6*A*). DMT and SMR transporters have previously been proposed to be evolutionarily related (129, 130). However, the SLC35/DMT and SMR structures also bear two notable differences. First, each domain of the SLC35 inverted repeat is composed of five TM helices. In the 3D structure, this pair of inserted helices pack against the membrane portal defined by helices 2_A_ and 2_B_ of the SMR transporters, sealing the portal and eliminating access to the substrate-binding site from the membrane (4). The second major difference between the SMR and SLC35/DMT structures is in the helix connectivity. Whereas in the SMRs, each transport domain is composed of a single, independently folded monomer; in the SLC35/DMT proteins, the transport domains do not correspond simply to the N- and C-terminal halves of the protein. The first transport domain is composed of helices 1, 2, 8, 9, and 10 and the second is composed of helices 3, 4, 5, 6, and 7 (Fig. 6*A*).

Although the possibility that the similarities between the SMR and SLC35/DMT folds arose *via* convergent evolution cannot be ruled out; structural correspondence between proteins with similar functions is usually considered evidence for evolutionary relatedness (131, 132). Moreover, we propose that the topological differences between the SMRs and SLC35/DMT folds can be plausibly explained by a divergent evolutionary pathway (Fig. 6*B*). This model posits an ancestral, dual topology transporter with the SMR fold and a TM helix insertion between TM helices 1 and 2. The introduction of this new TM helix would enforce a reorientation of TM helices 2, 3, and 4 relative to TM helix 1, disrupting the packing between TM helix 1 and the other TM helices in that monomer (Fig. 6*B*, panel 2). The 3D SMR fold could be preserved, however, by a domain swap during dimer assembly (Fig. 6*B*, panels 2 and 3) such that the now-inverted TM1 from the first monomer trades positions with TM1∗ in the opposite subunit and *vice versa* (Fig. 6*B*, panel 3). TM helix domain swaps have been observed in other membrane proteins (133, 134) and for engineered EmrE concatamers (29), and an analogous mechanism has been proposed for the evolution of the structurally similar, but topologically distinct, Pnu vitamin transporters and SWEET sugar exporters (135). Finally, a subsequent duplication/fusion of the 5-helix, domain-swapped ancestral dual topology transporter would give rise to the extant SLC35/DMT fold (Fig. 6*B*, panel 4). Duplication/fusion of 4-TM dual topology transporters are not unprecedented (27), but this process is more common for dual topology transporters with an odd number of transmembrane helices, since the N and C termini are on the same side of the membrane and connecting them does not require introduction of a transmembrane linker (28, 29). Although no sequence homology can be detected between the SLC35/DMT transporters and SMRs or even between symmetry-related helices of the SLC35/DMTs, lack of sequence homology is not uncommon in other families of evolutionary divergent transporters with shared folds (24, 136).

## Conclusions and perspective

The SMRs have provided a tremendously productive system for studying membrane protein evolution, transport mechanism, and microbial multidrug resistance. After 25 years of such studies, the SMR transporters finally joined the high resolution structural era in 2021. These recent crystal structures representing two of the four known functional subtypes have provided a platform for analyzing prior functional studies and understanding the structural features that contribute to substrate binding and transport for each SMR subtype: Gdm^+^, drugs, polyamines, and glycolipids. Moreover, structural homology with SLC35/DMT transporters suggests that, contrary to the proposal that the SMRs are unusual in having not evolved fused, inverted repeat architecture (29), it is probable that the SMR fold has indeed been preserved through this evolutionary mechanism, albeit with a helical insertion and domain swap along the way. These recent advances in understanding the molecular architecture bolster ongoing efforts to develop antimicrobials that target SMR proteins, either by inhibiting transporter assembly in order to sensitize bacteria to transported compounds (78, 137, 138) or by hijacking the nominal exporters to import antimicrobial compounds instead (139).

In addition, the molecular framework described here opens the door for future integrative functional, structural, and computational studies to understand how the SMR scaffold has been tailored to transport diverse substrates as the family has evolved. Such lines of inquiry are urgently important as bacteria continue to evolve around us. The Qac and Gdx subtypes, in particular, have found new roles in human-impacted environments, conferring bacterial resistance to household antiseptics (89), “dead-end” metabolites from the degradation of metformin (140) and other pharmaceuticals that accumulate in the environment or the human microbiome, and other agricultural and industrial chemicals (4). Genes encoding SMR transporters are currently spreading among bacterial populations, encountering new physiological contexts and substrate transport demands, driving co-selection of co-localized antimicrobial resistance genes in environmental reservoirs (76), and influencing microbial population compositions in the human microbiome and human-impacted environments (32, 76, 141), as the SMR transporters contribute to the ongoing story of natural selection at the human–microbe interface.

## Data availability

Sequences and annotation for SMR proteins identified in the bacterial GEBA genome set are available for download from the Deep Blue Data repository hosted by the University of Michigan with unique identifier doi.org/10.7302/0ynd-b343.

## Conflict of interest

The authors declare that they have no competing interests with the contents of this article.
